# Triaxiality and Plastic-Strain-Dependent Proposed PEAK Parameter for Predicting Crack Formation in Polypropylene Polymer Reservoir Subjected to Pressure Load

**DOI:** 10.3390/polym16152128

**Published:** 2024-07-26

**Authors:** Adam Kasprzak

**Affiliations:** 1 Department of Machine Design and Research, Wrocław University of Science and Technology, Łukasiewicza 7/9, 50-371 Wroclaw, Poland; adam.kasprzak@pwr.edu.pl; 2 Robert Bosch Sp. z o. o., Wrocławska 43, 55-095 Mirków, Poland

**Keywords:** polymer, polypropylene, triaxiality, crack, PEAK, PEEQ, TRIAX, plastic strain, reservoir

## Abstract

This article raises the topic of the critical examination of polypropylene, a key polymeric material, and its extensive application within the automotive industry, particularly focusing on the manufacturing of brake fluid reservoirs. This study aims to enhance the understanding of polypropylene’s behavior under mechanical stresses through a series of laboratory destruction tests and numerical simulations, emphasizing the finite element method (FEM). A novel aspect of this research is the introduction of the PEAK parameter, a groundbreaking approach designed to assess the material’s resilience against varying states of strain, known as triaxiality. This parameter facilitates the identification of critical areas prone to crack initiation, thereby enabling the optimization of component design with a minimized safety margin, which is crucial for cost-effective production. The methodology involves conducting burst tests to locate crack initiation sites, followed by FEM simulations to determine the PEAK threshold value for the Sabic 83MF10 polypropylene material. The study successfully validates the predictive capability of the PEAK parameter, demonstrating a high correlation between simulated results and actual laboratory tests. This validation underscores the potential of the PEAK parameter as a predictive tool for enhancing the reliability and safety of polypropylene automotive components. The research presented in this article contributes significantly to the field of material science and engineering by providing a deeper insight into the mechanical behavior of polypropylene and introducing an effective tool for predicting crack initiation in automotive components. The findings hold promise for advancing the design and manufacturing processes in the automotive industry, with potential applications extending to other sectors.

## 1. Introduction

The advent of polymeric materials, particularly polypropylene, first introduced to the market in the year 1951 [1], marked a significant milestone in the realm of material science and engineering. These materials have since played a pivotal role across a broad spectrum of industrial sectors. The selection of polymeric materials, especially polypropylene, is predicated on their exceptional mechanical properties, their inherent resistance to aging, their ability to maintain dimensional stability over the entirety of their lifecycle, their superior resistance to the initiation and propagation of cracks, and notably, their suitability for large-scale manufacturing processes such as extrusion and injection molding [2,3,4,5].

**Figure 1:**
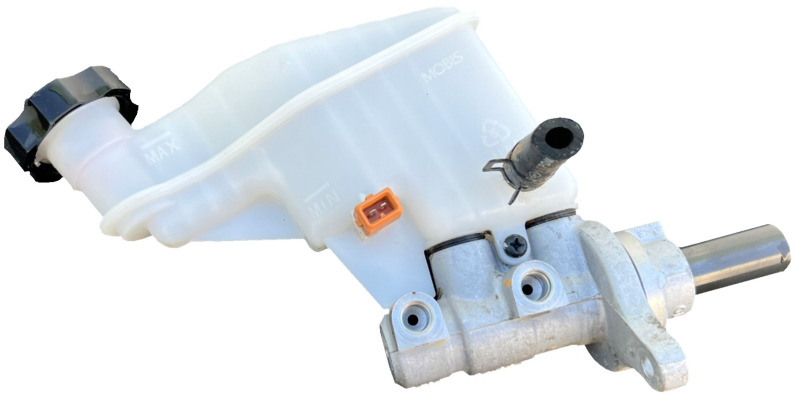
Brake fluid reservoir mounted on brake fluid pump.

Given these advantageous properties, polypropylene has been extensively utilized within the automotive industry, serving not only in the fabrication of interior vehicle components but also in the production of parts that are subjected to significant mechanical stresses and are integral to the safety of the vehicle occupants [6]. An illustrative example of such an application is the brake fluid reservoir (Figure 1), an essential component within the braking systems of passenger vehicles. This component is subjected to critical mechanical stresses during the initial factory filling process, a stage at which a specialized filling head applies a considerable multidirectional load on the filler neck, testing the material’s resilience.

In the context of the ongoing efforts towards the topological optimization of automotive components, including the aforementioned brake fluid reservoir, it is imperative to rigorously assess and validate the structural integrity of these components prior to initiating mass production. Historically, the adoption of numerical testing methodologies, with a particular emphasis on those based on the finite element method (FEM), has been favored. The preference for these methodologies stems from their ability to deliver rapid and accurate results, thereby facilitating the efficient design and optimization of components. However, the reliance on numerical methods for the comprehensive validation of component structures necessitates the utilization of highly accurate material models that faithfully represent the physical and mechanical behavior of the object under simulation [7].

The finite element method is a widely recognized numerical technique used in engineering and mathematical modeling to find approximate solutions to boundary value problems for partial differential equations. It is particularly known for its effectiveness in simulations involving complex geometries, diverse material properties, and various boundary conditions. However, FEM is not the only method available for such simulations and required specially treated elements in the crack region [8,9,10,11,12]. Alternative numerical techniques, such as the differential quadrature method (DQM) and the Bezier multi-step method, have also been developed and applied successfully, particularly in predicting crack initiation in polymers. These methods offer precise solutions for complex mechanical behaviors and can serve as suitable alternatives to FEM in specific scenarios. The differential quadrature method (DQM) is a numerical technique that approximates derivatives by a weighted sum of all function values in the domain. This method has been effectively applied in solving linear and nonlinear differential equations across various engineering fields. It is particularly noted for its high accuracy and efficiency in dealing with complex boundary conditions and geometries. The application of DQM in predicting crack initiation in polymers is discussed in [13]. This paper explores how DQM can be utilized to accurately predict the mechanical behavior of polymers, especially focusing on the challenges posed by cracks and defects. The Bezier multi-step method is another numerical technique that has been applied to the field of polymer simulations, particularly in predicting crack initiation. This method leverages the properties of Bezier curves, which are widely used in computer graphics and geometric modeling, to solve differential equations. By integrating the Bezier curve’s control points into the solution process, this method provides a flexible and accurate approach to modeling complex mechanical behaviors. The effectiveness of the Bezier multi-step method in this context is detailed in [14]. This article highlights the method’s precision and adaptability in handling the intricate dynamics involved in crack initiation and propagation in polymers. Both the differential quadrature method and the Bezier multi-step method offer promising alternatives to the finite element method for simulations involving complex mechanical behaviors, such as crack initiation in polymers. These methods provide precise, efficient solutions and can be particularly advantageous in scenarios where traditional FEM might face limitations due to geometric complexity, material heterogeneity, or boundary condition intricacies.

A series of tests have been carried out to determine the basic strength parameters of polymer materials, including determining the influence of triaxiality on the initiation of material cracking [15,16,17,18,19,20]. Importantly, all these studies mainly used printed samples, which allowed specially prepared shapes to be obtained, in turn allowing a relatively wide range of different states of material stress, presented as TRIAX (triaxiality), to be obtained. Due to the nature of producing brake fluid tanks through the injection process, it is not possible to obtain, among others, cylindrical samples at a reasonable cost. At the same time, the observed stress state in the tanks, i.e., the range from shear to triaxial stretching during the test of filling the tank with high-pressure fluid at a constant rate, allows us to focus on a narrowed range of material stress. Due to the above limitations and access only to existing brake reservoirs, the PEAK parameter (Adam Kasprzak’s plastic strain) was proposed, understood as a limit value sensitive to changes in the state of material strain (triaxiality), standardly used in such situations, called equivalent plastic strain (PEEQ). The standard approach also uses plastic strain energy as a crack indicator [21,22,23]. However, its determination requires complex material tests. Examples of such procedures are presented in [24]. Recent research suggests the benefits of using neural networks when investigating the mechanism of crack formation [25].

According to the presented literature review, the use of the proposed PEAK parameter is an intermediate solution between the simplest crack initiation models based only on the constant value of equivalent plastic strain PEEQ, and which can only be used with the participation of time-consuming and cost-intensive test procedures on samples specially prepared for this purpose. The use of the PEAK parameter, the calculation of which for a specific material requires only tests on existing components, is an opportunity to bypass restrictions in the availability of specialized samples while possibly reducing the unnecessary safety margin.

The purpose of further laboratory destruction tests and numerical simulations is to find the PEEQ and TRIAX parameters for the crack initiation locations, which will enable determining the boundary of the critical area and determining the original PEAK parameter, which is the limit of the safe range of the material state, guaranteeing consistency while minimizing the safety margin, which is important due to the production costs of the reservoirs.

## 2. Material Definition

All reservoirs used for further tests were made of Polypropylene called Sabic 83MF10 (provided by SABIC Poland Sp. z o. o.) without reinforcing inclusions such as glass fibers, etc. The basic strength parameters are presented in Table 1:

Due to the nature of the limited range of load characteristics, the numerical simulation used a material model taking into account the Young’s modulus 1200 [MPa], Poisson ratio 0.4 [−] and the Johnson–Cook plasticity model with the flow stress as a function of plastic strain $\sigma (\varepsilon_{p})$ characteristics:

(1) $$\sigma =25+21.3\cdot {\varepsilon_{p}}^{0.474}$$

## 3. Experiment Definition

The introduction to further research is the selection of 15 tanks, including 5 different models of tanks, 3 pieces each.

### 3.1. Laboratory Pressure Test

**Figure 2:**
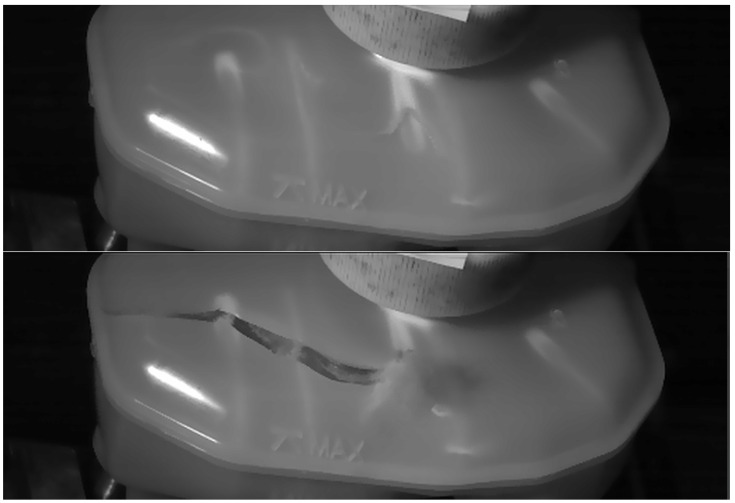
Crack propagation with average speed of 102 [m/s] (from connection of internal rib and out shell) on high-speed camera frames for reservoir no. 5.1.

**Figure 3:**
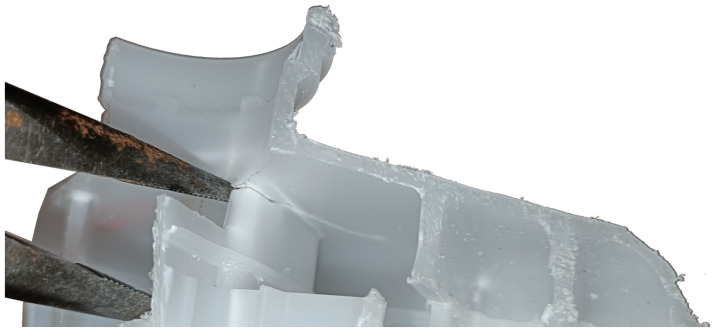
Crack for reservoir no. 5.2.

The first step to building the correlation was to perform burst tests using equipment that allowed for a constant increase in water pressure $∆p=1\left[bar/s\right]$ inside a tightly closed reservoir. During the above-mentioned test, the tank was immobilized only on its lower surface, which is normally attached to the brake fluid pump with horizontal metal pins, and the channels connecting the tank were plugged. The head applying pressure to the tank neck simulated the load applied during the tank filling process on the production line. The aim of this stage was to find the places of crack initiation, which was possible due to the speed of its propagation thanks to a high-speed camera (an example of two consecutive frames during which a crack occurs and develops is shown in Figure 2). An example of a crack location in a reservoir is shown in Figure 3

### 3.2. Simulation Analysis

**Figure 4:**
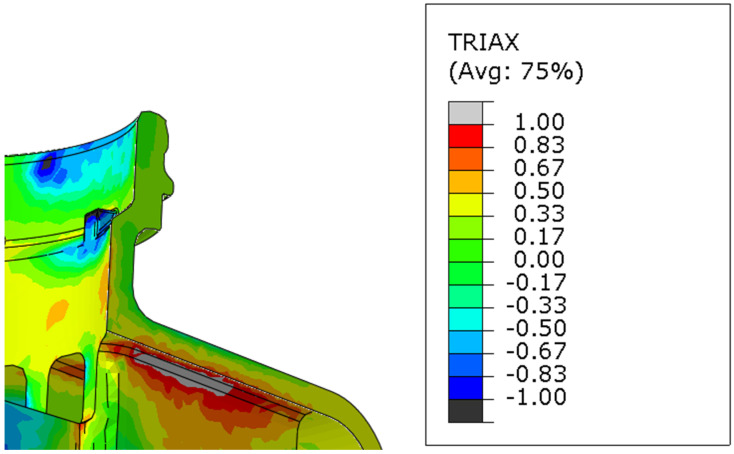
Triaxiality field for detected crack initiation spot in FEM analysis for reservoir no. 5.1.

The second step was to find PEEQ and TRIAX parameters at a large number of reservoir points, including crack-initiation spots and places that withstand the given load. Due to the repeatability of parameters in the laboratory test, it was reasonable to use static implicit simulation in Abaqus finite element method (FEM) software in our analysis. A virtual model was prepared using C3D10 a 10-node quadratic tetrahedron element with a global size of 1 [mm]. The results were read for centroidal element output position (example of triaxiality result is described on Figure 4).

## 4. PEAK Calibration

The methodology presented below is an original solution to the issue of including PEEQ and TRIAX parameters in a single PEAK parameter. The determined PEAK value is an individual limit for each material and test parameter (for which we perform calibration) beyond which crack initiation can be expected.

### 4.1. Data Comparison

For each of the reservoirs, the parameters PEEQ and TRIAX were read for the points where crack initiation occurred (nok) and where the applied load still did not cause damage, understood as a break in the continuity of the material (ok). This allowed us to isolate the safe operating range of the material. Due to the nature of the research and the state of current knowledge, it was important to look for points with the highest possible equivalent plastic strain PEEQ and Triaxiality TRIAX values. The places where the crack occurred (marked as nok in Table 2) were clearly determined based on observations with a high-speed camera during each laboratory test. Places marked as ok were designated based on the overall assessment of the reservoir and finding points with high values of both parameters.

### 4.2. Mathematical Formulation of PEAK

The PEAK curve proposed below, together with the parameter 0.37 [−], which is the limit value separating the places of crack initiation in the material from safe places, was determined on the basis of the previously presented data in accordance with the principle of minimizing the safety margin while maintaining all “nok” points above the proposed value.

(2) $${PEAK=PEEQ}^{\alpha }\cdot {\left(\frac{log\left|TRIAX+\beta \right|}{log\left|TRIAX+\gamma \right|}\right)}^{\delta }\cdot \frac{TRIAX}{\left|TRIAX\right|}$$

The proposed parts of the above equation have the following precisely defined aspects:

- ${PEEQ}^{\alpha }$—non-linear dependence on the basic parameter PEEQ;
- ${\left(\frac{log\left|TRIAX+\beta \right|}{log\left|TRIAX+\gamma \right|}\right)}^{\delta }$—asymptotic drop of isoline to the limit value for higher TRIAX;
- $\frac{TRIAX}{\left|TRIAX\right|}$—lack of sensitivity for points with negative TRIAX values, which automatically receive negative values of PEAK.

For testing SABIC 83MF10 material with the laboratory conditions mentioned previously, the PEAK parameter could be estimated as follows: α=0.7, β=1.0, γ=2, δ=0.5 and crack threshold ${PEAK}_{Sabic}=0.37$.

The final equation takes the following form:

(3) $${{PEAK}_{Sabic}=PEEQ}^{0.7}\cdot {\left(\frac{log\left|TRIAX+1\right|}{log\left|TRIAX+2\right|}\right)}^{0.5}\cdot \frac{TRIAX}{\left|TRIAX\right|}$$

**Figure 5:**
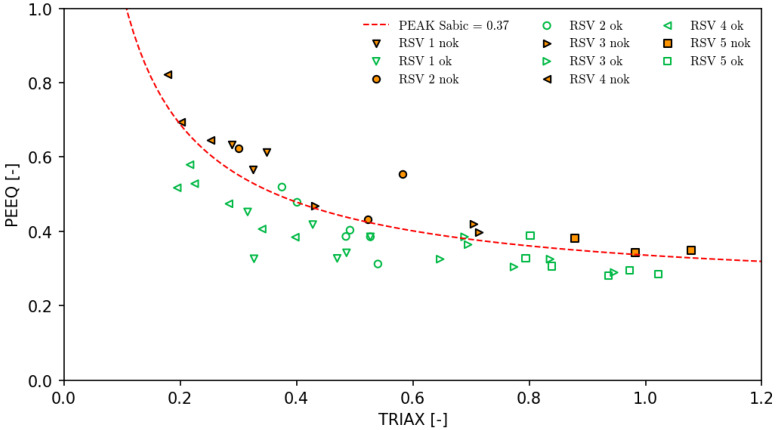
*PEAK_Sabic_* curve with raw data measured on reservoirs.

The raw data results, together with the final curve, define the limits; after these are exceeded, we can expect crack initiation, which can be visualized as below (Figure 5):

Due to the direct dependence of the PEAK parameter on the value of plastic strain PEEQ, its simple value results from the plasticity characteristics of the material. The lower the value of the yield strength and the flatter the yield curve, the faster the critical value of the PEAK parameter will be reached, suggesting the possibility of cracking. This is consistent with the standard use of a fracture mechanism called ductile damage when assessing the strength of polymeric materials, which depends linearly on the plastic strain to fracture value. The proposed PEAK parameter uses a similar characteristic, i.e., equivalent plastic strain PEEQ. The Young modulus value does not have a direct impact on the crack initiation moment suggested by the PEAK methodology, but may indirectly cause additional stresses at the border of areas with non-uniform load levels. The method and parameters for the Sabic material presented in the article were obtained for directly defined material parameters such as yield strength and Young modulus.

### 4.3. PEAK’s Prediction of Crack Initiation Pressure

The mere estimation of the limit curve, taking into account the minimum safe factor, is not a sufficient indicator of the ability of the proposed PEAK parameter to determine as close as possible to reality the pressure at which a tank filled with liquid will burst. It is important to determine the statistical percentage of simulation result utilization at the moment the PEAK parameter is exceeded (which is understood as the estimated moment of rupture) compared to the values obtained in the laboratory test. An additional checked function of the proposed parameter is its ability to predict the correct location of the first crack. For this purpose, data from laboratory tests were collected for five other types of reservoirs, three pieces each in Table 3.

Based on the data obtained, we can see that using the PEAK method, the following are true:

- The place of rupture was selected correctly in 66.7% of cases;
- The average utilization value reached 91.7% for all results and 90.8% excluding tank 8.3 from the analysis (due to a false positive result in this test);
- For 93.3% of tanks, the estimated result was obtained in the form of a lower result in the simulation than in laboratory tests with the highest possible utilization (close to 100% as possible).

**Figure 6:**
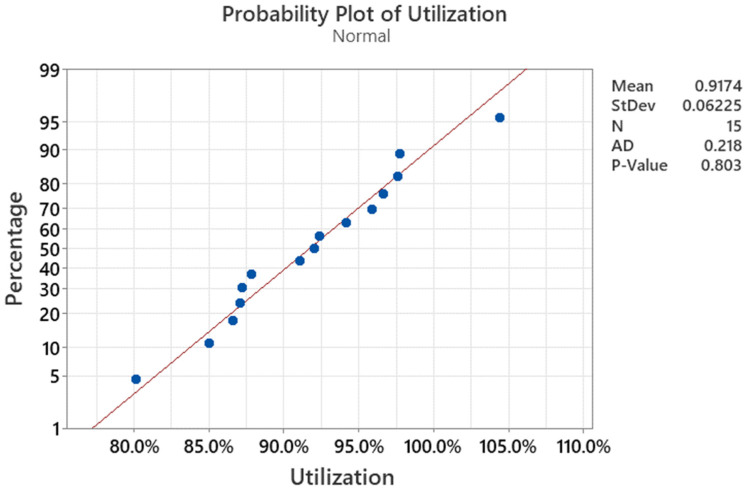
Utilization data fitting to normal.

**Figure 7:**
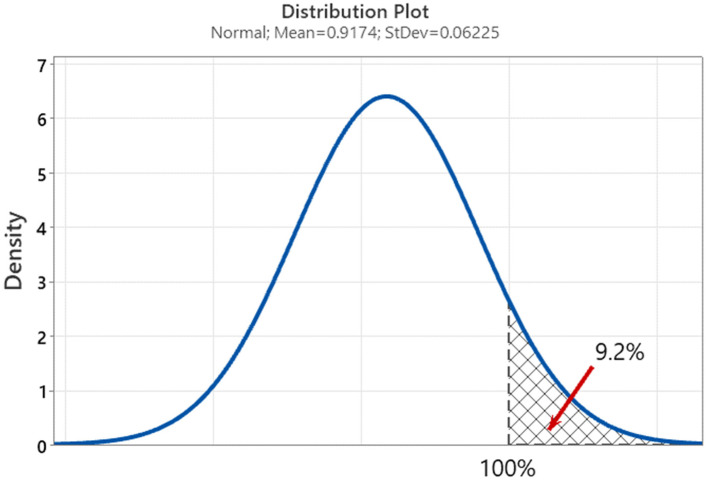
Utilization distribution plot using normal distribution with risk of simulation false positive result estimation.

**Figure 8:**
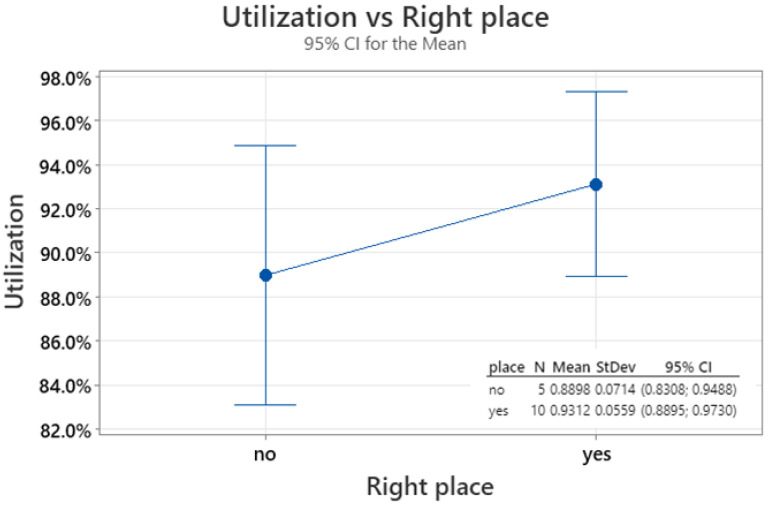
Utilization mean range acc. to right prediction of crack initiation place.

Statistical analysis (for graphic, see Figure 6, Figure 7 and Figure 8) estimated the crucial factors needed for making future predictions using the PEAK method:

- The obtained utilization results fit well into the normal distribution with parameters mean = 0.9174 and standard deviation = 0.06225, as indicated by the Anderson–Darling parameter = 0.218 and *p*-value = 0.803;
- The probability of a false positive simulation result compared to the laboratory result, forecast in accordance with the above parameters, is 9.2%;
- The analysis of variance for different utilization indications depending on the correct (or not) place of crack initiation showed statistically significantly different results, with a probability of approximately 76% (*p*-value = 0.237) (utilization for “yes”: mean = 93%, standard deviation = 5.6%, utilization for “no”: mean = 89%, standard deviation = 7.1%).

## 5. Conclusions

The comprehensive study presented in this article underscores the pivotal role of polypropylene, a versatile polymeric material, in the automotive industry, particularly in the manufacturing of critical components like brake fluid reservoirs. Through a meticulous examination of the material’s properties, coupled with rigorous laboratory destruction tests and numerical simulations, this research has successfully delineated the boundaries of critical areas for crack initiation in polypropylene components. The introduction of the PEAK parameter, a novel approach to assessing the material’s resilience under varying states of strain (triaxiality), represents a significant advancement in the field of material science and engineering. The findings from the burst tests and FEM simulations have provided invaluable insights into the behavior of polypropylene under mechanical stress, facilitating the identification of crack initiation sites and the determination of the PEAK parameter for the Sabic 83MF10 material. The calibration of the PEAK parameter, through a detailed analysis of PEEQ and TRIAX values at critical points, has enabled the establishment of a reliable criterion for predicting crack initiation. This criterion not only enhances the accuracy of structural integrity assessments but also optimizes the design process by minimizing the safety margin, thereby contributing to cost-effective manufacturing. The validation of the PEAK parameter’s predictive capability, as evidenced by the high degree of correlation between simulation results and laboratory tests, underscores its potential as a powerful tool for the automotive industry. The ability to accurately predict the location and pressure of crack initiation in polypropylene components can significantly improve the reliability and safety of automotive parts, ultimately benefiting manufacturers and consumers alike. In conclusion, this research has made a substantial contribution to the understanding of polypropylene’s mechanical behavior and its application in the automotive sector. The development and validation of the PEAK parameter mark a significant step forward in the optimization of material selection and component design. Future studies focusing on the application of the PEAK parameter to other polymeric materials and component geometries could further expand its utility, paving the way for advancements in material science and engineering that cater to the evolving needs of the automotive industry and beyond.

The choice of a constant pressure increase in laboratory tests was practical due to the requirements of a previously developed test procedure, standardly performed at Robert Bosch. The selected value of $∆p=1\;\left[bar/s\right]$ allows determining the specific pressure value at which the tank bursts without overestimating the results due to the physical inertia of the liquid and testing equipment, and clearly avoiding the significant impact of the creep and relaxation mechanism of the material from which the tank is made. The observations carried out so far allow us to conclude that an increase in the pressure rise rate (up to $∆p=3.5\;\left[bar/s\right]$) allows obtaining cracking pressure results that are 0.5 to 1 bar higher. Due to the limitations of the material model used to calibrate the PEAK method, the value obtained in this article retains its ability to correctly predict the moment of rupture only for the specifically indicated test procedure (including the speed of pressure increase in the tank). If the test procedure is changed, it is necessary to re-validate the PEAK parameters. No significant change in the nature of crack propagation was observed (also using a high-speed camera) due to the indicated range of pressure increase in the tank.

## 6. Discussion and Areas for Further Research

Further research should focus on increasing the universality of PEAK parameters by including in the material model used for tests characteristics such as:

- Dependence of Young’s modulus, yield strength and hardening curve on the strain rate;
- Taking into account the phenomena of material creep and relaxation and their impact on the formation of plastic deformations;
- Material anisotropy and its influence on the yield strength and Young’s modulus.

Taking into account these parameters will make the PEAK parameter results independent of the currently used test procedure.

The findings from this study on the utilization of polypropylene in automotive components, particularly focusing on the development and validation of the PEAK parameter, open several avenues for further research and discussion. The following topics could significantly contribute to the advancement of material science and engineering, especially in the context of polymeric materials:

- Material Variability and the PEAK Parameter: Investigating how variations in polypropylene formulations (including different molecular weights, copolymers, and composite materials) affect the PEAK parameter. This could help in refining the parameter for broader applicability across various polypropylene types.
- Comparison with Other Polymeric Materials: Extending the application of the PEAK parameter to other polymeric materials used in the automotive industry, such as polyethylene (PE), polyamide (PA) and other polypropylene (PP) blends from different suppliers.
- Economic Analysis: Performing a comprehensive economic analysis to quantify the cost savings achieved by optimizing component design using the PEAK parameter. This could also include assessing the impact on the manufacturing process, such as reduced material waste and improved production efficiency.
- Integration with Advanced Simulation Tools: Further integrating the PEAK parameter with advanced simulation tools and methodologies, such as machine learning algorithms, to enhance the predictive accuracy and efficiency of component design and optimization processes.

By addressing these topics, future research can build on the foundational work presented in this article, driving innovation in the use of polymeric materials in the automotive industry and beyond.

## Figures and Tables

**Table 1:** Basic material properties for Polypropylene Sabic 83MF10 [26].

Parameter	Value
Density	905 [kg/m^3^]
Tensile modulus	1200 [MPa]
Poisson ratio	0.4 [−]
Yield stress in RT	25 [MPa]

**Table 2:** Full data (PEEQ and TRIAX at specified points) for simulated and tested reservoirs (rsv).

RSV no	Result *	PEEQ [−]	TRIAX [−]
1.1	nok	0.613	0.348
	ok	0.452	0.316
	ok	0.385	0.527
1.2	nok	0.634	0.289
	ok	0.418	0.428
	ok	0.342	0.486
1.3	nok	0.566	0.325
	ok	0.327	0.470
	ok	0.326	0.327
2.1	nok	0.553	0.583
	ok	0.519	0.375
	ok	0.385	0.527
2.2	nok	0.445	0.523
	ok	0.478	0.401
	ok	0.386	0.485
2.3	nok	0.624	0.300
	ok	0.403	0.492
	ok	0.312	0.540
3.1	nok	0.487	0.432
	ok	0.364	0.695
	ok	0.325	0.647
3.2	nok	0.398	0.713
	ok	0.385	0.689
	ok	0.325	0.836
3.3	nok	0.419	0.705
	ok	0.289	0.946
	ok	0.304	0.774
4.1	nok	0.695	0.202
	ok	0.528	0.225
	ok	0.517	0.195
4.2	nok	0.645	0.253
	ok	0.579	0.217
	ok	0.474	0.284
4.3	nok	0.822	0.178
	ok	0.406	0.341
	ok	0.384	0.398
5.1	nok	0.349	1.079
	ok	0.281	0.936
	ok	0.295	0.973
5.2	nok	0.343	0.983
	ok	0.306	0.839
	ok	0.285	1.022
5.3	nok	0.381	0.879
	ok	0.327	0.794
	ok	0.388	0.802

* nok—crack nucleation place/ok—no crack nucleation place.

**Table 3:** Simulation and laboratory data comparison.

RSV No	Right Place *	Lab Result [bar]	Sim Result [bar]	Utilization
6.1	yes	17.8	17.4	97.8%
6.2	yes	18.9		92.1%
6.3	no	18.0		96.7%
7.1	yes	13.5	12.3	91.1%
7.2	yes	14.0		87.9%
7.3	yes	14.1		87.2%
8.1	yes	16.9	16.5	97.6%
8.2	no	17.2		95.9%
8.3	yes	15.8		104.4%
9.1	yes	10.5	9.7	92.4%
9.2	yes	10.3		94.2%
9.3	yes	11.2		86.6%
10.1	no	21.4	18.2	85.0%
10.2	no	22.7		80.2%
10.3	no	20.9		87.1%

* yes—right crack nucleation place prediction/no—wrong crack nucleation place prediction.

## Data Availability

The raw/processed data required to reproduce these findings cannot be shared at this time as the data also form part of an ongoing study acc. to grant Implementation PhD VI grant.
